# Molecular Mechanisms of Cerebrovascular Diseases

**DOI:** 10.3390/ijms23137161

**Published:** 2022-06-28

**Authors:** Anuska V. Andjelkovic, Richard F. Keep, Michael M. Wang

**Affiliations:** 1 Department of Pathology, University of Michigan Medical School, Ann Arbor, MI 48109, USA; 2 Department of Neurosurgery, University of Michigan Medical School, Ann Arbor, MI 48109, USA; rkeep@med.umich.edu; 3 Department of Molecular and Integrative Physiology, University of Michigan Medical School, Ann Arbor, MI 48109, USA; micwang@med.umich.edu; 4 Department of Neurology, University of Michigan Medical School, Ann Arbor, MI 48109, USA; 5 Neurology Service, VA Ann Arbor Healthcare System, Ann Arbor, MI 48105, USA

Cerebrovascular disease involves a range of conditions including ischemic and hemorrhagic stroke, vascular malformations, and vascular cognitive impairment and dementia (VCID). Cerebrovascular vascular disease has an enormous impact in terms of mortality, morbidity, and stress on families and societies. For example, stroke remains the second leading cause of death worldwide [1].

This Special Issue of the *International Journal of Medical Sciences* focuses on advances in our understanding of the molecular mechanisms underlying different types of cerebrovascular disease and subsequent brain injury. Treatments for cerebrovascular disease remain very limited (e.g., thrombolysis/thrombectomy for ischemic stroke) with many failed clinical trials. It is hoped that a greater understanding of the molecular events leading to cerebrovascular disease and its devastating consequences will help in the development of new therapies.

There have been great advances in our understanding of mechanisms leading to cerebral cavernous malformations (CCMs) with the discovery that loss-of-function mutations in three genes—*KRIT1/CCM1*, *CCM2*, and *PDCD10/CCM3*—lead to the inherited/familial forms of CCM. Phillips et al. [2] described how that discovery has led to a variety of CCM animal models that can be used to examine the molecular mechanisms underlying these malformations and to test potential therapeutic strategies.

The genetic basis of the most prevalent inherited form of stroke, cerebral autosomal dominant arteriopathy with subcortical infarcts and leukoencephalopathy (CADASIL), has also been identified: mutations in the protein NOTCH3. In this issue, Cartee et al. [3] explored the effects of cleaved products of NOTCH3 on the redox state of several extracellular polypeptides. Their study supports a novel mechanism whereby disulfide bonds of disparate proteins may be ruptured by interactions with pathological NOTCH3.

A common component of cerebrovascular disease is neuroinflammation. Choi et al. [4] reviewed the complex immune interactions that occur during stroke-induced brain injury and recovery both within the brain and peripherally. They then discussed how the peripheral immune system may provide new targets for treating the aftermath of stroke. Tulowiecka et al. [5] reviewed evidence indicating that lipoxin A4 is an important regulator of inflammation after stroke and provide evidence that the lipoxin A4 analog BML-111 has marked anti-inflammatory actions. As discussed by Sanchez and Rosenberg [6], there may also be an intersection between the inflammatory mechanisms induced by COVID-19 and stroke that may lead to stroke occurrence after Sars-Co-V2. They particularly focused on the role of peripheral macrophages and blood–brain barrier dysfunction.

As well as having an important role in acute stroke, inflammation has a crucial role in chronic cerebrovascular dysfunction, such as VCID. Tian et al. [7] reviewed the mechanisms by which chronic hypoperfusion can induce neuroinflammation in VCID and discussed potential therapeutic strategies. Hypertension also has effects on neuroinflammation. Hao et al. [8] reported hydrocephalus development in the spontaneously hypertensive rat (SHR) compared with the parent Wistar Kyoto rat strain. This was associated with inflammation at the choroid plexus (the blood–CSF barrier) and treating SHRs with minocycline reduced hydrocephalus and choroid plexus inflammation as well as improved cognitive function, white matter atrophy, and hippocampal neuronal cell loss.

Hydrocephalus can also occur in human subarachnoid hemorrhage (SAH) and animal models of SAH. Toyota et al. [9] examined the impact of aerobic capacity on such hydrocephalus. They found that rats with lower aerobic capacity had worse SAH-induced hydrocephalus. They posited that this was due to differences in iron handling and inflammatory mechanisms compared with rats with high aerobic capacity.

White matter damage occurs in both acute and chronic cerebrovascular disease and reducing such damage is an important therapeutic goal. Luo et al. [10] examined the impact of oligodendrocyte-specific deletion of the protein p53 in three models of white matter injury. In a cuprizone model of demyelination and white matter stroke, loss of p53 reduced brain white matter injury. However, in experimental autoimmune encephalitis (a model of multiple sclerosis), loss of p53 had no effect on spinal cord demyelination, suggesting potential location-specific effects of p53.

Current treatments for ischemic stroke include tissue plasminogen activator (tPA)-induced thrombolysis and thrombectomy. However, restoring cerebral blood flow can pose a risk of cerebral hemorrhage. Aamir et al. [11] examined the potential of using a heparin and arginine-based nanoformulation of plasmin for thrombolysis. Such nanoparticles increased plasmin stability and reduced stroke size in a mouse model without inducing overt cerebral hemorrhage.

Finding new treatments for cerebrovascular diseases is an ultimate research goal. As in all neurological conditions, developing therapeutic agents that will cross the blood–brain barrier is challenging, and this may contribute to the lack of success of many clinical trials for cerebrovascular disease. Nilles et al. [12] reviewed current evidence on the possibility of targeting organic anion and cation transporters present at the blood–brain barrier to enhance drug delivery.

A new therapeutic target in cerebrovascular disease, GPR39 (a member of the ghrelin family of G-protein coupled receptors), is reviewed by Xu et al. [13]. They discussed its role in brain and neurovascular homeostasis by maintaining excitatory/inhibitory tone in neural circuits as well as by controlling vascular and inflammatory tone.

An alternative to drug-based therapies may be cell-based therapeutics, such as neural stem cells (NSCs). Hamblin and Lee [14] reviewed the current preclinical evidence on the use of NSCs in cerebral ischemia, with a particular emphasis on its effects on neurovascular injury acutely after stroke.

Epigenetics is a fast-growing area of research in cerebrovascular diseases and targeting epigenetic changes may have an advantage by modulating multiple injury pathways. Demyanenko and Sharifulina [15] discussed current evidence on the role of acetylation and deacetylation in regulating signaling pathways and transcription factors in ischemic stroke. Sharifulina et al. [16] studied the role of two histone methyltransferases (SUV39H1 and G9a) and a DNA methyltransferase, DNMT1, in rat cerebral ischemia and found that G9a and DNMT1 may be therapeutic targets that reduce brain injury.

Overall, this Special Issue demonstrates the breadth of our advances in understanding the molecular mechanisms involved in cerebrovascular disease and subsequent brain injury. The goal for the future is how to translate that knowledge into improved treatments.
